# Supplementary material for: A New Circular Single-Stranded DNA Virus Related with Howler Monkey Associated Porprismacovirus 1 Detected in Children with Acute Gastroenteritis
Source: Viruses. 2022 Jul 4;14(7):1472. doi: 10.3390/v14071472 (PMC9319269; doi:10.3390/v14071472)
Supplement: Supplementary file 1 [file viruses-14-01472-s001.zip › viruses-1742633-supplementary.pdf]

Supplementary material:

A new circular single—stranded DNA virus related with Howler monkey associated porprismacovirus 1 detected in children with acute gastroenteritis

Fabiola Villanova [1], Flávio Augusto de Padua Milagres [2,3], Rafael Brustulin [2], Emerson Luiz Lima Araújo [4], Ramendra Pati Pandey [5], V. Samuel Raj [5], Xutao Deng [6,7], Eric Delwart [6,7], Adriana Luchs [8], Antonio Charlys da Costa [9‡] and Élcio Leal[1,‡,*]

[1]Laboratório de Diversidade Viral, Instituto de Ciências Biológicas, Universidade Federal do Pará, Belem, Pará, Brazil; 66075-000, Brazil; elcioleal@gmail.com (E.L.); fvillanova@gmail.com (F.V.)

[2]Secretary of Health of Tocantins, Palmas 77453-000, Tocantins, Brazil; flaviomilagres@uft.edu.br (F.A.d.P.M.); eu3rafael@gmail.com (R.B.)

[3]Public Health Laboratory of Tocantins State (LACEN/TO), Palmas 77016-330, Tocantins, Brazil.

[4]General Coordination of Public Health Laboratories of the Strategic Articulation Department of the Health Surveillance Secretariat of the Ministry of Health (CGLAB/DAEVS/SVS-MS), Brasília, Brazil; emerson.araujo@saude.gov.br (E.L.LA.)

[5]Centre for Drug Design Discovery and Development (C4D), SRM University, Delhi-NCR, Rajiv Gandhi Education City, Sonepat 131029 Haryana, India; ramendra.pandey@gmail.com (R.P.P.); deanacademic@srmuniversity.ac.in (V.S.R.)

[6]Vitalant Research Institute, 270 Masonic Avenue, San Francisco, CA 94118-4417, USA; xdeng@vitalant.org (X.D.); EDelwart@Vitalant.org (E.D).

[7]Department Laboratory Medicine, University of California San Francisco, San Francisco, CA 94143, USA

[8]Enteric Disease Laboratory, Virology Center, Adolfo Lutz Institute, São Paulo 01246-000, São Paulo, Brazil; driluchs@gmail.com (A.L.).

[9]Instituto de Medicina Tropical da Faculdade de Medicina da Universidade de São Paulo, São Paulo 05403-000, Brazil; charlysbr@yahoo.com.br (A.C.d.C.)

† These authors contributed equally to this work. ‡ These authors jointly supervised this work.

***** Correspondence: elcioleal@gmail.com

Figure S1

Howler monkey associated porprismacovirus 1 (Genbank ID: NC_026317)

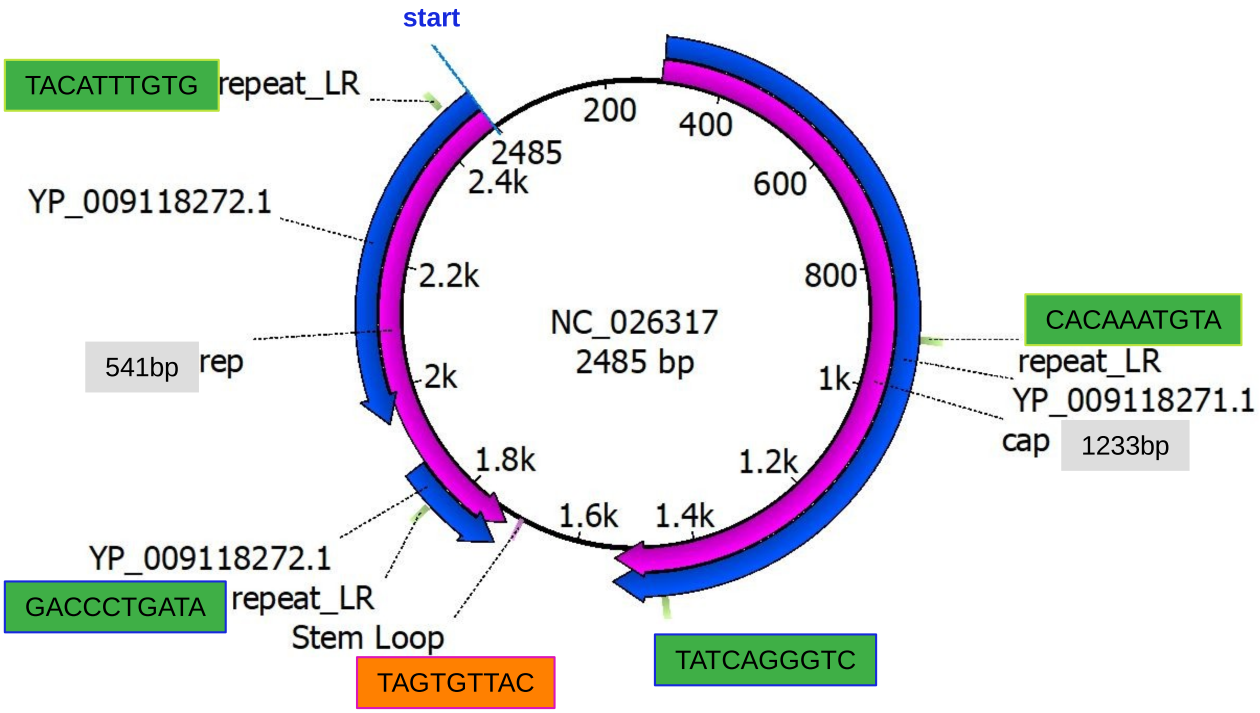

Figure S1. Genomic annotation of Howler monkey associated porprismacovirus 1 (Genbank ID: NC_026317). The sequence annotation indicated by blue bars were based on the original Genbank annotation, according to the predicted location of open reading frames of the reference sequence NC_026317 of CRESS-DNA viruses (Family: Cremevirales; Genera: Smacoviridae; species: Porprismacovirus). Blue bars indicate the annotation make in this study. Rep denotes the replicase protein and CAP denotes the capsid protein of these viruses. The sizes of Rep and Cap proteins are indicated in base pairs in the gray rectangles. The TAGTGTTAC loop is indicated in an orange rectangle. Inverted repeats are indicated in green rectangles. The blue line, perpendicular to the circular genome indicate the first nucleotide of the sequence.

Figure S2

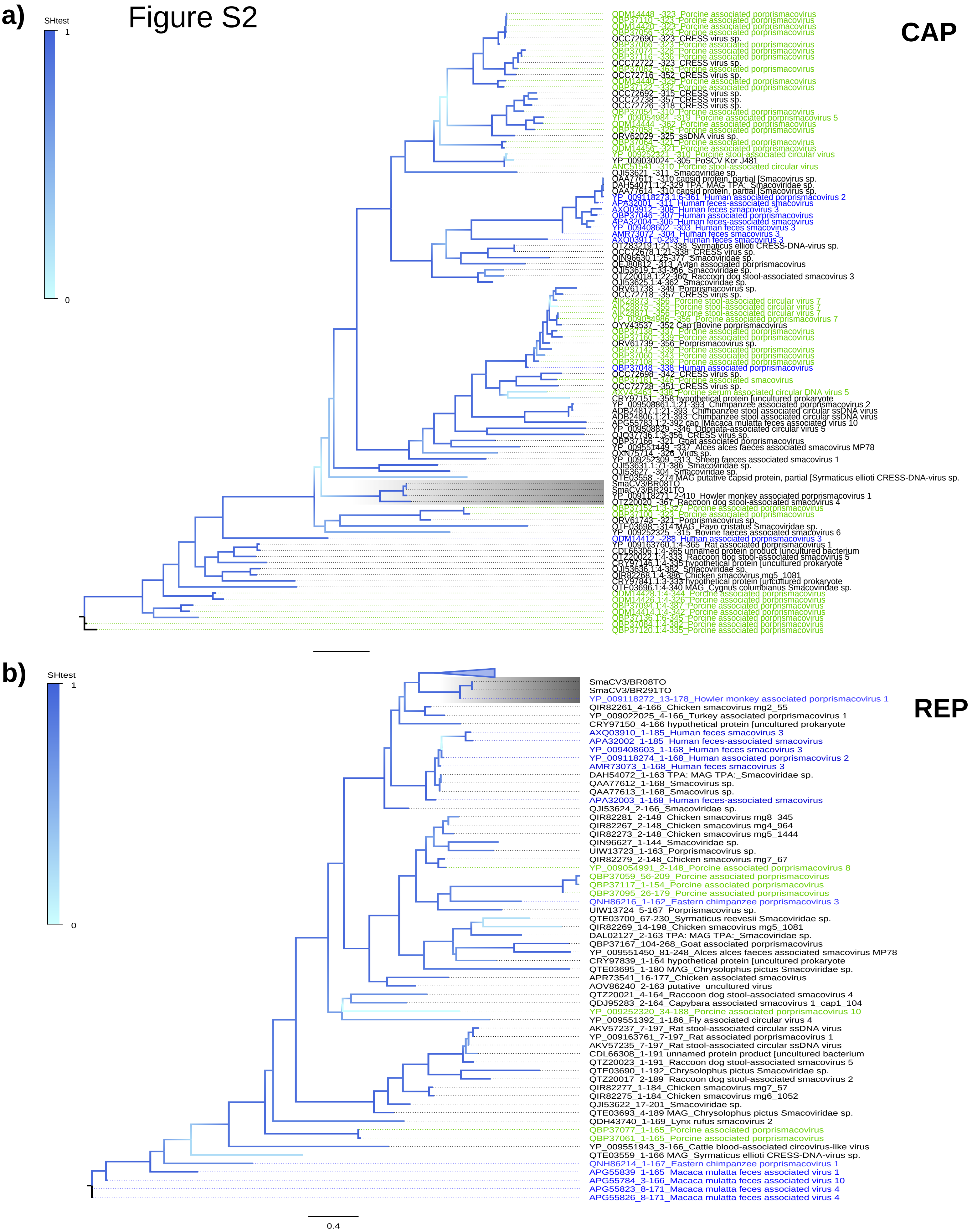

Figure S2. Phylogenetic trees of CAP and REP of smacoviruses. a) Tree inferred with the predicted CAP protein of CRESS-DNA viruses. B0 Tree inferre with the predicted REP protein of CRESS-DNA viruses. Maximum likelihood trees inferred assuming the JTT model. The tree is mid-rooted and the statistical branch support (bases on the Shimodaira-Hasegawa test) is indicated in a blue color scaled. Lower horizontal bar indicate the amino acid substitutions per site. Porprismacovirus identified in this study are indicated by gray rectangle.
